# Epiboly generates the epidermal basal monolayer and spreads the nascent mammalian skin to enclose the embryonic body

**DOI:** 10.1242/jcs.180703

**Published:** 2016-05-01

**Authors:** Eleni Panousopoulou, Carl Hobbs, Ivor Mason, Jeremy B. A. Green, Caroline J. Formstone

**Affiliations:** 1 Department of Craniofacial Development and Stem Cell Biology, Guys Tower, Kings College London, London SE1 1UL, UK; 2 Wolfson-CARD, Kings College London, London SE1 1UL, UK; 3 MRC Centre for Developmental Neurobiology, New Hunts House, Kings College London, London SE1 1UL, UK

**Keywords:** Epidermal morphogenesis, Progenitor monolayer, Epiboly, Celsr1, Cell polarity

## Abstract

Epiboly is a morphogenetic process that is employed in the surface ectoderm of anamniotes during gastrulation to cover the entire embryo. We propose here that mammals also utilise this process to expand the epidermis and enclose the body cavity and spinal cord with a protective surface covering. Our data supports a model whereby epidermal spreading is driven by the primary establishment of the epidermal basal progenitor monolayer through radial cell intercalation of a multi-layered epithelium towards the basal lamina. By using a suspension organotypic culture strategy, we find that this process is fibronectin-dependent and autonomous to the skin. The radial cell rearrangements that drive epidermal spreading also require ROCK activity but are driven by cell protrusions and not myosin II contractility. Epidermal progenitor monolayer formation and epidermal spreading are delayed in *Crash* mice, which possess a dominant mutation in Celsr1, an orthologue of the core planar cell polarity (PCP) *Drosophila* protein Flamingo (also known as Stan). We observe a failure of ventral enclosure in *Crash* mutants suggesting that defective epidermal spreading might underlie some ventral wall birth defects.

## Abstract (precis)

**Summary:** The nascent mammalian epidermis spreads to enclose the embryo trunk through a process akin to epiboly, which has important implications for human birth defects of the abdominal wall.

## INTRODUCTION

Epiboly is one of a series of complex tissue movements that shape the basic body plan of species of amphibian and fish embryos (Kopsch, 1895; Holtfreter, 1943). In amphibians, epiboly is defined as the thinning and spreading of ectodermal and mesodermal progenitors (Keller, 1975; Wilson and Keller, 1991). Keller described the thinning process as one of radial intercalation (Keller, 1978), where two cell layers intermingle cell-on-cell to form one cell layer only, followed by planar cell shape changes. Each stage drives tissue spreading. The process of epiboly contributes therefore to expansion of the embryo surface during early development.

In mammals, the surface ectoderm of the developing embryo transforms, through a process of tissue stratification, into the skin (epidermis), which protects our bodies from mechanical, chemical and microbial insult. Stratification of the epidermis can be visualised in the mouse embryo from around embryonic day (E)13.5 (depending on mouse strain) and is marked by the appearance of the suprabasal layer (Koster and Roop, 2007). Delamination of basal progenitors appears to initiate suprabasal layer formation (Williams et al., 2014b), whereas later stage suprabasal cells are generated by asymmetric cell divisions within the progenitor monolayer (Lechler and Fuchs, 2005; Williams et al., 2014b). As the suprabasal cell layer becomes established, a regular array of hair follicle placodes punctuate the basal monolayer and begin a process of organised down-growth, the orientation of which is steadily tail-ward. The striking alignment of oriented hair follicle down-growth across the developing epidermis is a consequence of planar cell polarity signalling operating within the basal progenitor monolayer (Devenport and Fuchs, 2008).

Although we understand the embryonic origins of the skin (Fuchs, 2007), very little is known about how the epidermal progenitor layer (basal monolayer), which builds the protective surface barrier of the body, actually forms. Here, we report that the primary establishment of the basal monolayer is coupled to an early spreading of the epidermis that eventually encloses the embryo body. We demonstrate that this process mirrors epiboly of the ectoderm during gastrulation in amphibians. Our data suggest a model whereby progenitor monolayer formation is driven by radial cell intercalation. As in *Xenopus*, we find that epidermal thinning and spreading is dependent on fibronectin but also requires ROCK activity and actin-rich lamellipodial protrusions, but not myosin II contractility. Epidermal spreading is disrupted in the Celsr1 mouse mutant *Crsh* and correlates with defects in ventral closure of the embryonic body in this mutant. These findings argue that the mammalian embryonic skin encloses the embryo through a morphogenetic strategy utilised by anamniotes to spread their surface ectoderm and provide new insights into the underlying basis of abdominal wall defects.

## RESULTS

### Epidermal progenitor monolayer formation correlates with dorso-ventral spreading of the nascent epidermis to enclose the embryonic body

**Fig. 1.**
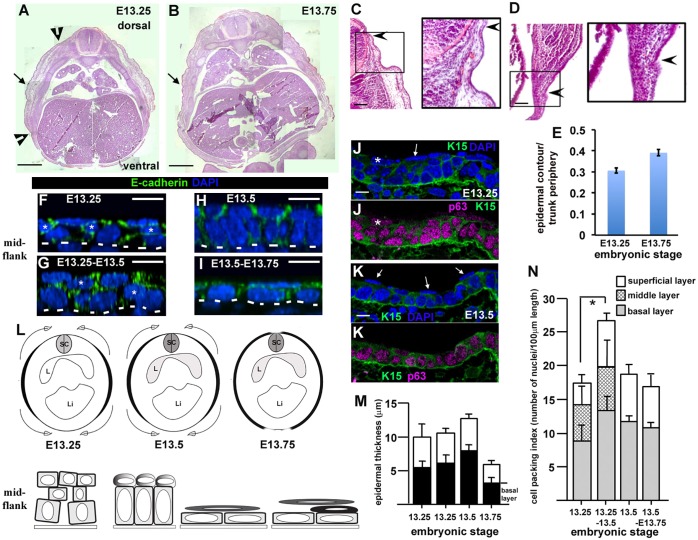
**Epidermal basal monolayer formation correlates with spreading of the surface ectoderm to enclose the embryonic body.** (A,B) Stitched images of wild-type transverse mid-flank trunk paraffin sections stained with H&E. Internal organ landmarks (i.e. lungs) were used to ensure similar anterior-posterior positions were analysed, *n*>3 embryos analysed for each stage. Black arrowheads (E13.25) indicate contour extent of flank ectoderm. Black arrows mark region analysed in whole-mount immunostaining (see F–I). Scale bars: 0.5 mm. (C,D) Details of wild-type E13.25 transverse sections indicating examples of the dorsal (C) and ventral (D) interfaces of the surface ectoderm. Panels are equivalent to those points marked by black arrowheads in A. Black arrows demonstrate how the epidermal interfaces were visualised. Scale bars: 100 µm. (E) Fractional epidermal spreading calculated as a ratio of epidermal contour length to section periphery (mean±s.d.). *n*>3 embryos for each stage. (F–I) Representative *z*-stack images taken from whole-mount immunostained skins dissected from the mid-flank. Scale bars: 10 µm, *n*>3 embryos for each stage. Asterisks show middle cells; the dotted lines represent the basal lamina. (J,K) Representative confocal images (*n*>3 embryos) of immunostained frozen sections. Arrows label p63-negative periderm cells. K15 denotes cytokeratin-15 staining (Troy et al., 2011; see also Fig. S2M). Scale bars: 10 µm. (L) Schematics of epidermal epiboly. Top panel and bottom panel for each embryonic stage represent the contour length of the epidermis (in black) and epidermal morphology, respectively. Top panel, embryo is represented in transverse section, with dorsal to the top (SC, spinal cord; L, lung; Li, liver). Bottom panel, an initial multi-cell layered immature epidermis locally generates a progenitor monolayer with an overlying superficial layer by E13.5, which takes on a squamous morphology between E13.5 and E13.75. Suprabasal cells (black) appear just prior to E13.75. The described cellular changes (bottom panel) correlate with tissue spreading (top panel). (M) Histogram of epidermal thickness through the stages indicated. (N) Histogram of cell packing in mid-flank of embryos fixed at the stages indicated showing disappearance of the intermediate layer by E13.5. *P<0.05 (two-tailed *t*-test). For M,N, measurements were taken from six images for each of three embryos at each stage and results are mean±s.d.

To investigate early epidermal development we analysed wild-type mouse embryos staged between E13.25–E13.75 when the epidermis had not yet stratified. We examined hematoxylin and eosin (H&E)-stained wax-embedded transverse sections and observed several intriguing features along the dorso-ventral extent of the E13.25 embryonic body surface that had disappeared by E13.75. H&E staining was most intense in the embryo flank (see contour within black arrowheads, Fig. 1A; enlarged views, Fig. 1C,D). Flank ectoderm also covered a thicker mass of underlying tissue when compared to dorsal and ventral surfaces (see surface contour within black arrowheads Fig. 1A; enlarged views, Fig. 1C,D). The interfaces between flank tissue and dorsal and ventral tissue were easily discerned (black arrowheads, Fig. 1A,C,D) and were used to measure the contour length of the surface ectoderm (Fig. 1E). By E13.75, the surface ectoderm appeared to mostly enclose the embryo body (Fig. 1B,E). Taken together, these data are consistent with a spreading process from the mid-flank (upper panel, Fig. 1L) which was confirmed in transverse frozen sections (Fig. S1A–C). The latter also revealed that flank ectoderm did not fully enclose the embryo body even by E14 (Fig. S1B). Our findings therefore reveal a hitherto unrecognised morphogenetic process in the mid-gestation mammalian embryo: the dorsal and ventral enclosure of the embryonic body surface. This is an important period of development and correlates with an increase in the circumference of the embryonic trunk but not its anterior-posterior (forelimb-to-hindlimb) length (Fig. S1D).

To examine the morphology of the surface ectoderm during the spreading process we analysed mid-flank tissue (black arrows, Fig. 1A,B) by whole-mount immunostaining (Fig. 1F–I). Between E13.25 and E13.5 the surface epithelium progressively thickened (Fig. 1M). A disordered multi-cell layered epithelium (Fig. 1F,G,L), comprising a basal layer of cells sitting on the basal lamina, a middle layer of cells (asterisks, Fig. 1F,G) and a layer of superficial cells, transformed into an orderly monolayer of columnar basal progenitor cells overlain with rounded superficial cells (Fig. 1H,L). We also observed patches of progenitor monolayer that were ‘naked’ with no overlying superficial cells (Fig. S2K,K′). Quantification of cell packing index (i.e. the number of cells per unit area) revealed a significant increase in basal layer cell packing (i.e. density of cells sitting on the basal lamina) between E13.25–E13.5 and disappearance of the middle layer of cells by E13.5 (Fig. 1N). Thus, spreading of the surface ectoderm correlated with the disappearance of a middle cell layer and epidermal progenitor monolayer formation (Fig. 1L). From E13.5 both the basal cells and overlying superficial cells changed shape and became squamous (Fig. 1I,L). These planar cell shape changes were reflected by a decrease in tissue and basal layer thickness (Fig. 1M) suggestive of a second phase of spreading following basal monolayer formation.

**Fig. 2.**
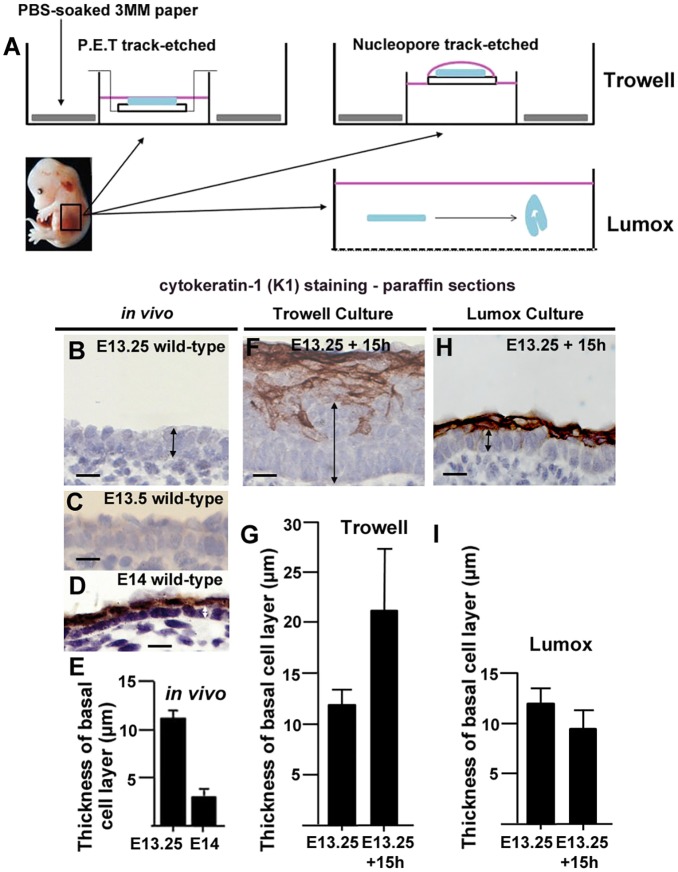
**Trowell versus Lumox culture.** (A) Experimental setup for Trowell (two variations) and Lumox culture (see Materials and Methods). (B–D) Wax sections of E13.25 to E14 wild-type epidermis *in vivo* (*n*=3 for each stage). (F,H). Wax embedded sections of indicated cultured explants. Scale bars: 10 µm. (E,G,I) Mean±s.d. basal layer thickness measurements of epidermis *in vivo* (E) and indicated explant types at *t*=15 h (G,I). At least 20 measurements were taken on at least three independent explants for each condition. Lack of K1 staining was used to identify and measure basal layer thickness (black vertical arrows indicate examples of measurements).

Subsequent staining with markers of basal progenitors confirmed the emergence of superficial periderm from E13.25 (Fig. 1J,K; Fig. S2A,B). Suprabasal cells appeared during the squamous phase at around E13.75 and were marked by cytokeratin-1 (Fig. 2B–D; Fig. S2E). We consistently distinguished a period of 9–12 h between the onset of surface ectoderm spreading at E13.25 and the appearance of epidermal suprabasal cells around E13.75 (*n*>8 litters). Taken together, therefore, our data suggest that epidermal progenitor layer formation is coupled to surface ectoderm spreading and dorsal and ventral enclosure of the embryonic body.

### An epidermal progenitor monolayer forms in Lumox culture but not Trowell culture

Our data revealed that formation of the skin progenitor monolayer correlated with the conversion of a disordered multi-cell layered surface epithelium (hereafter immature epidermis) into an orderly basal monolayer with overlying squamous periderm. This transformation correlated with spreading of the embryo surface and was strikingly reminiscent of epiboly in *Xenopus* (Keller, 1980). To test the similarities between amphibian epiboly and early mammalian epidermal morphogenesis, we turned to organotypic (*ex vivo*) culture. We made explants from wild-type flank and cultured them for 15 h, at which point the epidermis had begun stratification and expressed cytokeratin-1 (K1, also known as KRT1; Torchia et al., 2013). We used K1 to distinguish between basal progenitor cells and suprabasal cells in wax-embedded sections (Fig. 2B–D, Fig. S2E,F). We first tested variations on the well-established Trowell culture (Trowell, 1959; Fig. 2A) but explants formed a substantially thickened K1-negative basal layer, indicating that these explants did not reflect the *in vivo* situation (Fig. 2D,E versus F,G). Instead, we developed an alternative culture system. Reasoning that both the filter and the surface tension of the liquid film might interfere with normal morphogenesis, we turned to a suspension approach using Lumox dishes, which have a gas-permeable bottom to enable improved oxygenation (Fig. 2A). Flank epidermis for Lumox explants was peeled away from the underlying mesoderm but the dermis was left *in situ*. These suspension explants reliably formed a basal monolayer (seen in ten of ten samples), albeit columnar and not squamous (compare the thickness of the basal layer in Fig. 2D,E versus H,I), which was overlain by proliferative suprabasal cells as indicated by K1 and Ki67 staining in wax sections (Fig. 2H, Fig. S2G,I). Lumox culture therefore recapitulated the *in vivo* situation (see also Fig. S2K–N), and we considered it a suitable organotypic culture method to study formation of the epidermal basal monolayer.

### Autonomous epidermal spreading in skin explants is not associated with planar cell divisions or a decrease in cell packing

**Fig. 3.**
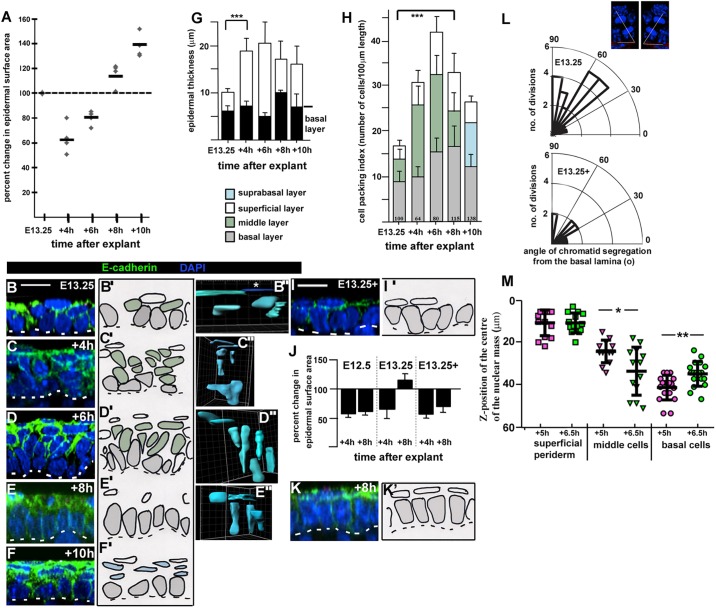
**Epidermal spreading is driven by radial intercalation.** (A) Scatter plot of epidermal surface area changes in different explants after the indicated number of hours in culture, generated from two different wild-type litters. 100% (dashed line) indicates no change. Horizontal black lines indicate mean values. (B–F) Representative *z*-stack images of E13.25 explant layer index. Images were taken in the centre of the explants, *n*=3 explants for each time-point. (B′–F′) Camera Lucida images of B–F. Grey cells are basal layer cells, green cells are middle cells, white cells are superficial periderm cells and blue cells are proliferating suprabasal cells. (B″–E″) 3D reconstructions of live cell shapes over time in Lumox culture. Surfaces were created manually using Imaris (Bitplane) Surpass view. (B″) Asterisk denotes a squamous periderm cell, labelled dark blue for clarity. Three explants were analysed for each time point. Scale bars: 10 µm. The dotted lines represent the basal lamina. (G) Histogram of changes in epidermal thickness over timecourse shown in A. Results are mean±s.d. ***P<0.0001 (two-tailed *t*-test). (H) Histogram of changes in cell packing in explants over the timecourse shown in (A). Bars are colour coded as images in B′–F′. Results are mean±s.d., *n*>3 explants from each time point. Average percentage change in surface area for each time point (A) is indicated above the *x*-axis. ***P<0.0001 (two-tailed *t*-test against basal layer packing). (I,I′,K,K′) As B–F′ with *n*=3 explants. (J) Histogram of explant spreading at *t*=4 h and *t*=8 h, comparison of *n*=3 explants for each stage taken at E12.5, E13.25 and E13.25+3–4 h. Results are mean±s.d. (L) Polar plots of angle of chromatid segregation from the basal lamina. Telophase divisions: the mean angle of their oriented cell division was measured relative to the basal lamina (see insets, top right). Insets are representative examples of the methodology used. (M) Scatter plots showing relative *z*-position of the nuclear mass of the same live cells at E13.25+5 h (magenta) and E13.25+6.5 h (green) with respect to the top of the superimposed *z*-stack images. Black lines indicate mean±s.d. A total of 44 GFP-expressing cells were analysed, *n*=14 superficial periderm cells, *n*=12 middle cells and *n*=18 basal cells. *P<0.05; **P<0.005 (two-tailed *t*-test).

Our data suggested that the tissue-spreading process that encloses the embryonic body was coupled to epidermal basal monolayer formation. To test this hypothesis further we established a spreading assay in Lumox culture by examining changes in the surface area of E13.25 explants after various times in culture. This revealed that explant surface area consistently declined in the first 4 h of culture (*n*=4 timecourse experiments) but then recovered (4–6 h) and eventually spread beyond its original size after 8–10 h in culture (Fig. 3A).

Reduced surface area in Lumox explants correlated with an increase in layer index from, on average, 2.79 to 4.9 and with significant tissue thickening (Fig. 3C,G). As surface area decreased, we found that the index of cell packing (the number of cells per unit area) in the middle layer increased substantially with a more modest increase evident in the superficial layer (Fig. 3H), despite a phospho-histone H3 (PH3) index of less than 5% (Fig. S3C), and a high cell-death index in the superficial periderm (Fig. S3E). Taken together, these data suggest that the increases in epidermal thickness and middle layer and superficial layer (periderm) packing resulted from cell rearrangement although cell proliferation must also play a role. Importantly, basal cell packing hardly changed, indicating that planar cell shape changes did not contribute to surface area reduction. It seems most likely therefore that both basal cells and middle cells were displaced superficially, thus thickening the middle and superficial periderm layers during this period. Three-dimensional reconstruction of live cells from explants in which we had induced a mosaic expression of membrane GFP (mGFP), further supported this hypothesis (Fig. 3B″–E″).

A full recovery and an increase in surface area beyond their original size was consistently observed in explants by 8 h (*n*=4 timecourse experiments) and correlated with the formation of a columnar basal monolayer (Fig. 3E–E″,H). At 8 h in culture we found areas of ‘naked’ basal monolayer (Fig. S2L,L′) with no overlying superficial periderm cells, as observed *in vivo* (Fig. S2K,K′), supporting the hypothesis that Lumox culture recapitulates progenitor monolayer formation in the embryo.

Spreading of explants beyond their original size (6–8 h in culture; Fig. 3A) was largely at the expense of middle layer packing, whereas basal layer packing progressively increased (Fig. 3H). The latter ruled out cell flattening as a mechanism for tissue spreading over this time period. Rather it suggested that from 4 h to 8 h in culture, spreading was linked to an increased number of cells in the basal layer. We reasoned that there were two possible explanations for the increase in basal cell packing; horizontal cell divisions within, or radial intercalation of middle cells into, the basal layer. Cell proliferation was less than 5% during explant culture (Fig. S3C) and, importantly, very few basal layer telophase divisions were horizontal (below 45° to the basal lamina; top panel, Fig. 3L) suggesting that most cell divisions contribute new cells to the middle layer. Our data argues therefore that between 4 and 8 h in culture, cell rearrangements most likely drive surface area recovery and expansion. To test this hypothesis further, we undertook live imaging of E13.25 Lumox skin explants expressing mosaic mGFP. We made *z*-stack images at two time points, E13.25+5 h and +6.5 h, when explants were exponentially spreading (Fig. 3A). Registration of the same cells was achieved by marking the explant surface with the dye DiI. Superimposition of *z*-stacks and three-dimensional reconstruction of individual cell shapes (*n*=44) revealed that middle layer cells moved basally and basal layer cells became columnar (Fig. 3M; Movies 1–3) consistent with our model of radial intercalation. Taken together, the data argue against surface shedding of middle cells as explants recover surface area. Superficial or periderm cells remained at the tissue surface, arguing against any significant contribution to the basal layer (Fig. 3M). Therefore, our findings point to cell rearrangement as the primary mechanism of tissue autonomous basal monolayer formation and associated epidermal spreading.

### Explants lacking a middle epithelial layer do not spread

To further test the requirement for a middle cell layer, we performed a similar timed analysis on explants from slightly older embryos (roughly 3–4 h older; E13.25+), when the middle layer had mostly disappeared (Fig. 3I,I′, Fig. S3B). We predicted that the absence of a middle layer would prevent spreading. We found that these later explants behaved as the earlier stage explants (Fig. S3A,B,D,F) except that they recovered little of their surface area and did not expand beyond their original size (Fig. 3J) despite establishing an orderly basal monolayer after 8 h (Fig. 3K,K′) and despite the presence of horizontal telophase divisions within the basal layer during the culture period (Fig. 3L). Explants taken at E12.5, when the surface ectoderm exists as a primitive monolayer (Devenport and Fuchs, 2008), also did not spread (Fig. 3J). Taken together, these data demonstrate that the autonomous spreading potential of the surface ectoderm is limited to E13.25 and further argues that the presence of middle cells drives surface area recovery and expansion.

### A second phase of epidermal spreading is associated with cell shape changes

Maximal spreading of E13.25 explants occurred at 10 h in culture (Fig. 3A) and was associated with a change in cell shape from columnar to cuboidal (Fig. 3F,F′), quantified as a decreased basal layer thickness (Fig. 3G) and a decreased cell packing index (Fig. 3H). Notably, planar cell shape changes following radial intercalation of a multi-cell layered epithelium define epiboly in amphibian embryos (Keller, 1980). From 12 h in culture, basal cells returned to a columnar morphology as described above (Fig. 2G).

Taken together, the explant experiments reveal a crucial period between E13.25 and E13.5 during which time an orderly epidermal progenitor monolayer is formed. Live imaging of cell behaviour over time supports a vertical (radial) cell intercalation mechanism in which middle layer and basal layer cells of the disordered multi-cell layered surface epithelium generate the basal monolayer. A mechanism of radial intercalation explains how the surface area of the embryonic surface ectoderm expands during this first phase of spreading when basal cells become more orderly and increasingly well packed (Fig. 1L). Our explant experiments additionally reveal a second phase of spreading associated with planar cell shape changes. *In vivo* we found that epidermal basal progenitors converted into a squamous morphology as ventral enclosure progressed around the embryo body (Fig. 1L). It is possible that planar cell shape changes dominate this second phase of spreading. In our model of radial intercalation-driven basal monolayer formation, cell packing increases in the basal layer from E13.25 to E13.5, which should potentiate tissue spreading in this second phase.

### Failure of epidermal spreading is linked to ventral enclosure defects in the Celsr1 mouse mutant *Crsh*

**Fig. 4.**
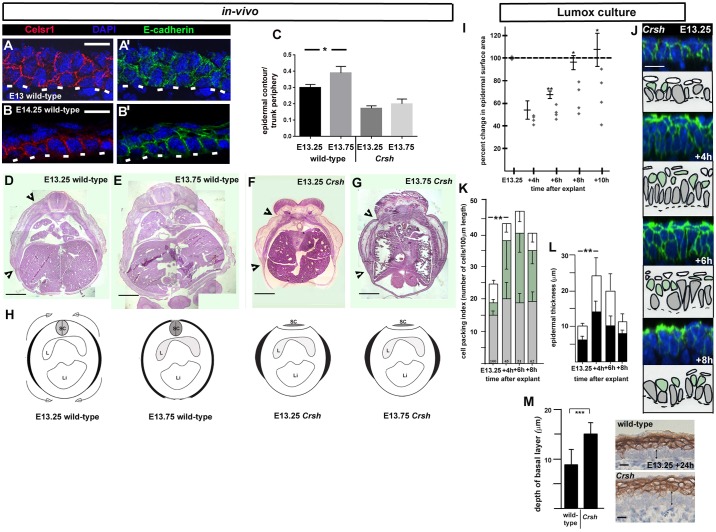
**Epidermal spreading and basal monolayer formation is dependent on Celsr1.** (A–B′) Celsr1 immunofluorescence staining of indicated stages. Scale bars: 10 µm. Transverse mid-flank epidermis, frozen sections, *n*=3 embryos. White dashed lines in A–B′ mark the basal lamina. (C) Fractional epidermal spreading calculated as a ratio of epidermal contour length to section periphery. Results are mean±s.d., *n*=3 embryos for each stage. *P<0.05 (two-tailed *t*-test). (D–G) Stitched images of wild-type (D,E) and *Crsh* (F,G) transverse mid-flank trunk paraffin sections stained with H&E. Black arrowheads indicate contour extent of nascent epidermis. Scale bars: 0.5 mm. By E13.75 the epidermis has mostly enclosed the embryonic body in wild-type (E) but has failed to spread in *Crsh* homozygotes, *n*=3 for each stage. (H) Schematics of epidermal epiboly in wild-type and its failure in *Crsh* homozygotes, black areas represent contour length of the epidermis for each embryonic stage. Embryos are represented in transverse section, with dorsal to the top (SC, spinal cord; L, lung; Li, liver). The flattened spinal cord in *Crsh* represents an early stage neural tube defect. (I) Scatter plot of explant spreading in *Crsh* littermates, bars to the left of each time point show the mean±s.d. of wild-type littermates, *n*=3 for each time point. *P<0.05; **P<0.005 (two-tailed *t*-test). (J) Representative *z*-stack images of E13.25 explant layer index. Images were taken in the centre of the explants, *n*>3 for each time point. Camera Lucida images are shown below. Grey cells are basal layer cells, green cells are middle cells, white cells are superficial periderm cells. The dotted lines represent the basal lamina. Scale bar: 10 µm. (K) Histogram of changes in cell packing in *n*>3 explants over the timecourse shown in I. Bars are colour coded as Camera Lucida images in J. Results are mean±s.d. The mean percentage change in surface area for each time point (I) is indicated above the *x*-axis. **P<0.001 (two-tailed *t*-test). (L) Histogram of changes in epidermal thickness over timecourse shown in I. Results are mean±s.d. **P<0.001 (two-tailed *t*-test). (M) Average basal layer thickness measurements of indicated explants at *t*=24 h. Results are mean±s.d. from at least 20 measurements on at least three independent explants for each condition. K1 staining was used to identify and measure basal layer thickness (black vertical arrows in images indicate examples of measurements). ***P<0.0001 (two-tailed *t*-test). Scale bars: 10 µm.

Our explant assays support an epiboly-type mechanism for the epidermal spreading process associated with dorsal and ventral body enclosure. Molecular studies in teleosts (zebrafish) have implicated the Celsr family of adhesion G-protein-coupled receptors in epiboly (Carreira-Barbosa et al., 2009). Celsr1 is expressed in the developing epidermis where it exhibits a restricted expression to the basal monolayer (Devenport and Fuchs, 2008; Fig. 4B,B′). In the immature epidermis, we found that it was expressed in a thickened multi-cell layered epithelium (Fig. 4A,A′), consistent with our model where middle layer and basal layer cells intercalate to generate the epidermal basal monolayer.

We tested a role for Celsr1 in epidermal epiboly by analysing an ENU-induced mouse, possessing a dominant mutation in *Celsr1*, named *Crash* (*Crsh*; Curtin et al., 2003). Examination of transverse H&E-stained wax-embedded sections revealed that ventral enclosure failed at E13.75 in *Crsh* homozygotes when compared to wild-type littermates (seen in seven out of seven examined; Fig. 4C–H; Fig. S1E). Timing of mammary gland formation was similar between *Crsh* mutants and wild-type littermates (data not shown) ruling out any developmental delay. To test whether the failure of ventral enclosure correlated with defective epidermal spreading, we performed a timecourse of *Crsh* flank skin explant spreading in Lumox culture. We found that despite a predominance of horizontal cell divisions in the basal layer (Fig. S3G,I), *Crsh* homozygote explants were significantly less able to recover their surface area from 4 h to 10 h in culture compared to wild-type littermates (Fig. 4I). Notably, the middle layer did not disappear and *Crsh* explants did not form a basal monolayer (Fig. 4J–L) even after 24 h in culture (Fig. 4M) after which time they remained less well spread than their wild-type littermates (data not shown). Notably, despite the thickened basal layer at 24 h, the *Crsh* suprabasal cell layer appeared similar to wild-type, indicating that defective basal monolayer formation did not disrupt suprabasal cell generation (Fig. 4M).

The data reveal therefore that the failure of *Crsh* epidermis to spread is autonomous to the skin and argues for the associated failure of ventral enclosure *in vivo* being intrinsic rather than, for example, an indirect effect of the axial shortening phenotype of the *Crsh* embryo (Curtin et al., 2003). Taken together, these data confirmed the association between primary establishment of the progenitor monolayer and epidermal spreading. Furthermore, the failure of epidermal spreading directly correlated with failure of ventral enclosure in *Crsh*.

### Failure of epidermal spreading and ventral enclosure in E13.75 *Crsh* homozygotes was not associated with a thicker immature epidermis or aberrant cell division index and orientation

**Fig. 5.**
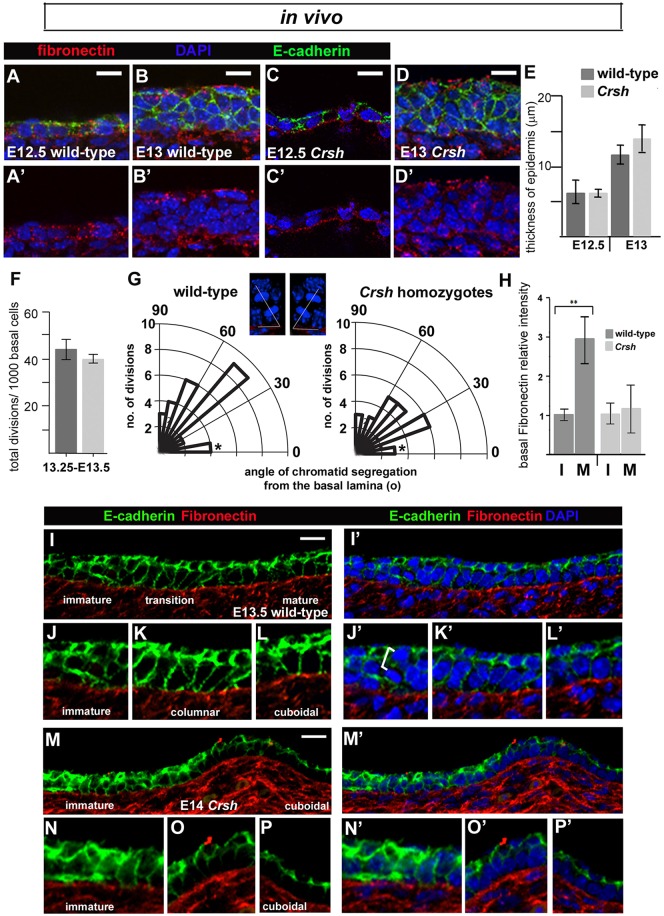
**Progenitor layer formation in *Crsh* is associated with aberrant ECM deposition.** (A–D′) Immunostaining of transverse flank epidermis, frozen sections. Scale bars: 10 µm. Representative examples of epidermal thickening and multi-cell layered epithelium formation in wild-type (A–B′) and *Crsh* (C–D′) littermates. (E) Quantification of epithelial thickness in two *Crsh* litters staged at E12.5 and two staged at E13 (four wild-type and four *Crsh* homozygote embryos). Results are mean±s.d. (F) Frequency of cell division within the immature epidermis. Results are mean±s.d. Three wild-type and three *Crsh* embryos from two litters were analysed. (G) Polar plots of telophase divisions in E13.25–E13.5 wild-type (*n*=25) and *Crsh* (*n*=25) epidermis were scored and the mean angle of their oriented cell division was measured relative to the basal lamina (*x*-axis). Asterisks highlight the cell divisions in the 0–15° binned group, which were planar and occurred only within the superficial layer. Insets are representative examples of the methodology used to measure the mean angle of cell division relative to the basal lamina (see Materials and Methods). White lines denote angles of cell division measured. Three wild-type and three *Crsh* embryos from two litters were analysed. (H) Relative fibronectin staining pixel intensities at the basal lamina of the immature (I) normalised to the mature (M) epithelium in wild-type (*n*=3) and *Crsh* littermates (*n*=4). Results are mean±s.d. **P<0.014 (*t*-test). (I–P′) Fibronectin and E-cadherin immunostaining of longitudinal flank epidermis, frozen sections. The E-cadherin channel (green) has been digitally brightened to show cell outlines. Scale bars: 10 µm. Representative examples of progenitor monolayer formation in wild-type (I–L′) and *Crsh* (M–P′) littermates. J–L′ and N–P′ show enlargements of images from I,I′ and M,M′ highlighting the progression in progenitor monolayer formation.

Cell packing analyses of the immature epidermis in *Crsh* homozygotes (E13.25; Fig. 4J,K versus Fig. 3H) together with examination of frozen sections from E12.5–E13 embryos (Fig. 5A–E; Fig. S4A–C) argue that the thickened surface epithelium apparent in the mutant at E13.75 *in vivo* (Fig. 4G) did not result from an earlier thickening of the surface epithelium in *Crsh* homozygote skins compared to wild-type. We found no significant differences between the timing and extent of *Crsh* epidermal thickening and no significant differences in their respective mitotic indices (Fig. 5F). Notably, the distribution of telophase division angles within the E13.25–E13.5 basal layer was shifted towards horizontal in *Crsh* homozygotes (Fig. 5G). The observed predominance of vertical divisions in wild-type was consistent with our findings in E13.25 Lumox explants (Fig. 3L).

### Lack of Celsr1 and Fz6 planar polarisation during epidermal basal monolayer formation

Celsr1 is an orthologue of *Drosophila* protein Flamingo (also known as Stan), a key component of the core planar cell polarity (PCP) pathway (Usui et al., 1999). PCP organises the alignment of cellular structures, for example, the wing hairs of insects and the hair follicles of mammals, in the plane of the epithelium, along particular body axes (reviewed by Devenport, 2014). The study of Carreira-Barbosa et al. (2009) suggested that the role of Celsr in teleost epiboly was distinct from its later functions in PCP signalling. We found no evidence for planar polarisation of either Celsr1 or Fz6 (also known as FZD6) protein distribution until the mature basal monolayer was well established at E13.75 (Fig. S4F–I). We also observed that the timing of basal monolayer formation was similar in *fz6*-knockout embryos and wild-type littermates (Fig. S4J,K) despite clear later stage planar polarisation phenotypes in the skin of the homozygote mutant (Guo et al., 2004). We concluded that, as for teleost epiboly, epidermal spreading is not tightly coupled to planar cell polarisation.

### Basal extracellular matrix deposition is aberrant in *Crsh* homozygotes during progenitor monolayer formation

Both epidermal spreading and ventral enclosure fail in *Crsh* homozygotes by E13.75. Because epidermal spreading directly correlated with epidermal basal monolayer formation, we assessed the latter in *Crsh* littermates. We examined immunostained frozen sections and observed that in wild-type littermates, the wild-type basal monolayer formation was non-uniform (i.e. occurred in patches, with regions of mid-flank epidermis most advanced). This regional pattern explained the variability in basal layer cell packing from E13.25 to E13.5 *in vivo* (Fig. 1N) and enabled us to capture, in a single image, the progression of epithelial thinning from a multi-cell layered epithelium to a columnar and then cuboidal basal monolayer (Fig. 5I–L′). We used markers of cell–cell adhesion (E-cadherin) and cell–matrix adhesion (fibronectin) to assess both epidermal morphology and organisation. These analyses demonstrate that the transition from a columnar to cuboidal basal monolayer occurs *in vivo* as expected for an epiboly-like mechanism. Furthermore, they provide further support for the hypothesis that our E13.25 Lumox explant cultures recapitulate the temporal progression of basal monolayer formation and maturation.

In wild-type, we were also struck by the increasing enrichment of fibronectin at the boundary between epidermis and underlying dermis (basal lamina) (Fig. 5H,I). Vinculin (a cytoplasmic actin-binding protein enriched in basal membrane focal adhesions; Peng et al., 2011) mirrored fibronectin enrichment (Fig. S4D,D′) whereas laminin was uniformly enriched at the basal lamina (data not shown). Conversely, where we found basal monolayer formation in *Crsh* homozygote epidermis (Fig. 5M–P′), fibronectin staining was highly variable between embryos and litters (see error bars in Fig. 5H) and vinculin staining showed the same pattern as fibronectin (Fig. S4E,E′). We also observed abnormal fibronectin deposition in the surface epithelium of E12.5–E13 *Crsh* homozygotes compared to wild-type, with the homozygote mutants exhibiting fibronectin deposition at both superficial and basal interfaces (compare Fig. 5D′, Fig. S4C with Fig. 5B′, Fig. S4B). Taken together, we conclude that deposition of fibronectin within the immature epidermis was defective in *Crsh* homozygotes.

### Epidermal spreading depends upon integrin–fibronectin interactions at the basal lamina

**Fig. 6.**
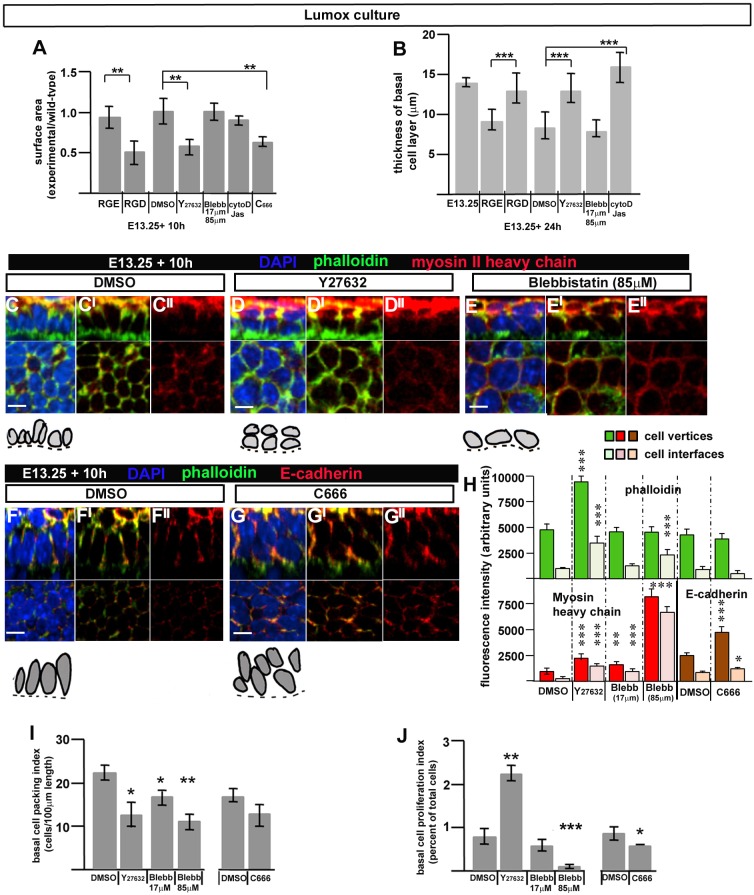
**Progenitor monolayer formation and epidermal spreading in Lumox culture depends upon integrin–fibronectin interactions at the basal lamina, ROCK activity and cell protrusivity.** (A) Histogram of surface area of explants after indicated treatments after 10 h in culture. Surface area is mean of experimental explants, *n*>3, relative to mean value of wild-type explants, *n*>3, from same experiment. Error bars are standard deviations. **P<0.001 (two-tailed *t*-test). (B) Quantification of basal layer thickness in paraffin sections of *n*=3 explants after indicated treatments after 24 h (see also Fig. S2N–V). Results are mean±s.d. ***P<0.0001 (two-tailed *t*-test). (C–G″) Top panels are *z*-stack (*z*-plane) images of whole-mount immunostained explants, *n*>3 in each case. Bottom panels are ‘en-face’ views (*xy*-plane). Scale bars: 10 µm. Camera Lucida images of basal layer cells (grey) sitting on the basal lamina (dashed line) are shown underneath each immunostained image. (H) Histogram of fluorescent intensities at cell vertices or cell interfaces (*n*>25 for each fluorescent label). (I) Quantification of planar packing in the basal layer of treated explants. The *P*-value was 0.053 for C666 treatment. (J) Quantification of PH3 cell proliferation index in the basal layer of treated explants. In H–J, results are mean±s.d. *P<0.05; **P<0.005; ***P<0.0005 (two-tailed *t*-test, for comparison of experimental with control explants). At least three explants were analysed.

Epiboly in *Xenopus* is also demonstrably fibronectin-dependent (Marsden and DeSimone, 2001). The aberrant fibronectin deposition in the *Crsh* mutant, particularly at the basal lamina, suggested that fibronectin might have a functional role in epidermal spreading in common with the role of basal fibronectin in *Xenopus* ectoderm epiboly. To test this experimentally, we cultured suspension explants with Arg-Gly-Asp (RGD) peptides, which mask integrin-binding sites on fibronectin, thereby inhibiting integrin–fibronectin interactions and adhesion (Hautanen et al., 1989). We tested both epidermal spreading and basal monolayer formation. RGD-treated explants in Lumox culture did not recover their surface area as observed in wild-type littermates or RGE control explants (Fig. 6A). Staining for the suprabasal marker K1 showed that in these RGD-treated explants, the K1-negative basal layer was significantly disorganised and thickened (Fig. 6B; Fig. S2R,T), whereas explants from littermates incubated with the non-inhibitory RGE peptide (Hautanen et al., 1989) formed a K1-negative basal monolayer (Fig. S2Q) as observed in wild-type (Fig. 4M). This indicates that, as in *Xenopus* epiboly, integrin–fibronectin interactions at the basal lamina are required for basal monolayer formation and epidermal spreading, consistent with our model of radial intercalation where middle cells increase contact with the (fibronectin-containing) basal lamina and move basal-ward, increasing the occupancy of the basal layer to drive epidermal spreading.

### Epidermal spreading requires ROCK activity and cell protrusivity but not actomyosin contractility

The data above argue that, as in frogs and fish, the surface epidermis of mammals spreads through Celsr- and fibronectin-dependent epiboly. The failure of epidermal spreading in *Crsh* homozygotes, which also exhibit neural tube defects (Curtin et al., 2003), prompted us to investigate signalling downstream of Celsr1, which had not been previously examined for teleost epiboly (Carreira-Barbosa et al., 2009). Nishimura et al. (2012) recapitulated failure of neural tube closure in an avian model by disrupting both *Celsr1* and Rho kinase (ROCK) activity. Equally compelling were reports that mouse mutants in ROCK1 and ROCK2 display abdominal wall defects (Shimizu et al., 2005; Thumkeo et al., 2005). To test whether ROCK might link Celsr1 to cell rearrangements driving epidermal spreading and thus ventral enclosure, we treated E13.25 wild-type explants with Y-27632 (17 µM), a ROCK inhibitor. We found that ROCK inhibition disrupted epidermal spreading (Fig. 6A) and basal monolayer formation (Fig. 6B, Fig. S2U) when compared to DMSO-treated controls (Fig. 6A,B; Fig. S2P). We next asked whether ROCK activity was linked to actomyosin contractility, as reported in the avian neural tube (Nishimura et al., 2012). Treatment with Blebbistatin specifically inhibits this process (Cai et al., 2010) but did not disrupt basal monolayer formation or epidermal spreading (Fig. 6A,B; Fig. S2V) despite a significant reduction in cell division when used at higher concentrations (Fig. 6J). Conversely, ROCK-inhibited explants increased their basal cell proliferation index (Fig. 6J) but still failed to spread (Fig. 6A). Both ROCK-inhibited and Blebbistatin-treated explants decreased basal cell packing (Fig. 6I, compare Fig. 6C–C″ with Fig. 6D–D″ and Fig. 6E–E″) but only the latter spread beyond their original size. These findings support a direct correlation between basal monolayer formation and explant spreading and are consistent with our model of radial intercalation.

ROCK inhibition specifically elevated actin levels (as determined by phalloidin staining) at both cell vertices and cell interfaces (Fig. 6H). Actin-based cell protrusive behaviour drives the cell rearrangements associated with convergent extension movements during amphibian gastrulation (Elul et al., 1997), mammalian neural tube formation (Williams et al., 2014a) and cell rearrangements associated with morphogenesis of the mammalian limb ectoderm (Lau et al., 2015). Explants treated with cytochalasin D or jasplakinolide, which respectively destabilise or stabilise actin filaments and would be expected to inhibit cell movements, failed to form a basal monolayer (seen in five out of five explants, Fig. 6B; Fig. S2S) but their surface area did not reduce significantly (Fig. 6A). Taken together, these data suggested a total failure of cell movement throughout the culture period. Thus, active cell rearrangements likely drive both explant surface area reduction (form 0–4 h in culture) and explant surface area recovery and/or expansion (from 6–8 h), consistent with our earlier timecourse analyses (Fig. 3). Arp2/3 promotes formation of branching actin filaments during lamellipodia formation as well as cadherin-associated cell–cell junctions (reviewed by Rotty et al., 2013; Abu Taha et al., 2014). Inhibition of actin-based cell protrusions using the Arp2/3 inhibitor (C666) resulted in failure of basal monolayer formation and surface area recovery (Fig. 6A,F–G″). Notably, the level and distribution of phalloidin staining were undisturbed (Fig. 6H). Cell proliferation was reduced (Fig. 6J) but so was planar packing (Fig. 6I). Importantly, E-cadherin levels were significantly elevated particularly at cell vertices (Fig. 6H), suggesting disturbed cell adhesion. We concluded that epidermal spreading requires both ROCK activity and actin-based cell protrusions, which most likely promote radial cell rearrangements to generate the skin progenitor monolayer and thus drive the spreading process and ventral enclosure.

## DISCUSSION

Progenitor epithelia are essential building blocks for tissue and organ construction. The findings of this study argue that the progenitor monolayer epithelium (basal monolayer) of the mammalian skin is established through a morphogenetic process akin to epiboly in anamniotes that concomitantly spreads the surface ectoderm of the mammalian embryo to enclose the body. In anamniotes (frogs and fish), epiboly expands the ectoderm to cover the entire embryo at gastrulation stages. Epiboly-like cell intercalation of definitive endodermal cells has also been reported during mouse gastrulation (Kwon et al., 2008). We propose that mammalian embryos also utilise this process to rapidly enclose themselves in a protective epidermis during a much later stage of development and that failure or delay in epidermal enclosure could lead to ventral closure defects including those of the abdominal wall.

Both *in vivo* and *ex vivo* we find that basal monolayer formation correlates with the disappearance of a middle cell layer from the multi-cell layered epithelium of the surface ectoderm. Our explant experiments support a model for radial intercalation of middle cells towards the epidermal basal lamina as the cellular basis for basal monolayer formation. The importance of epidermal spreading for establishing an orderly basal monolayer is further supported by the failure of both these processes in the Celsr1 mouse mutant, *Crash*. Importantly, radial intercalation of middle cells into the basal layer would contribute new basal cells during an important period of circumferential growth of the embryo body (Fig. S1D) and thus ensure expansion of the surface ectoderm at this time. Radial intercalation offers the embryo a rapid route to increasing basal layer occupancy. Indeed, it is unlikely that horizontal cell divisions could alone achieve the required increase in basal cell numbers between E13.25 and E13.5. Furthermore, our *ex-vivo* data suggest that cell division maintains a middle layer of cells between E13.25–E13.5. We do expect, however, that horizontal cell divisions within the established progenitor monolayer directly contribute to tissue spreading between E13.5 and E13.75.

As demonstrated for epiboly in teleosts (Carreira-Barbosa et al., 2009), we found that epidermal spreading is dependent on Celsr1. Our analyses of the Celsr1 mutant, *Crsh*, revealed defects in ventral enclosure which might correlate with abdominal wall defects observed in this mutant (Murdoch et al., 2014). Malformations of the abdominal body wall are a common human birth defect (Blazer et al., 2004) but their aetiology is unclear. Animal models for ventral body wall formation are rare, which has hindered understanding of its embryology and of the aetiology of human ventral body wall defects. Although mouse genetic models are available they have been little studied (Brewer and Williams, 2004). Murdoch et al. (2014) have reported genetic interactions between multiple mouse mutants exhibiting abdominal wall defects. We reveal that defective ventral enclosure in one mutant, *Crsh*, is directly linked to defective epidermal spreading. It is interesting that another of the mutants studied was in the *Drosophila* apicobasal polarity component Scribble (Murdoch et al., 2014) and that Scribble-knockout mice (Carnaghan et al., 2013) exhibit abdominal wall defects. Notably, both *Crsh* homozygotes and Scribble mutants exhibit the severe neural tube defect, craniorachischisis (Curtin et al., 2003; Murdoch et al., 2003). Furthermore, we reveal that ROCK, which acts downstream of Celsr1 in other tissue systems (Nishimura et al., 2012), also regulates epidermal epiboly. It is highly compelling, therefore, that ROCK1 and ROCK2 mutant mice display abdominal wall defects (Shimizu et al., 2005; Thumkeo et al., 2005), lending support to the hypothesis that defects in epidermal spreading might be an underlying cause.

In addition to being Celsr1- and ROCK-dependent, epidermal spreading requires actin-based lamellipodial-type protrusions but not acto-myosin contractility. PCP protein dysfunction in amphibians results in defects in the polarity and stability of lamellipodia and the subsequent failure of the planar polarised cell rearrangements, which drive convergent extension (Wallingford et al., 2000; Wallingford, 2012). The cell-protrusion-based convergent extension movements of teleost fish also depend on ROCK (Marlow et al., 2002). Taken together, these findings suggest that the morphogenetic processes that either spread or elongate vertebrate tissues are similar at the tissue, cell and molecular scale (despite their progression along different body axes). It is noteworthy that in amphibians, where all gastrulation movements are interdependent, disruption of PCP signalling can influence both epiboly and convergent extension movements (Ossipova et al., 2015). In teleost fish, however, mutations in Vangl2 and Frizzled perturb convergent extension but do not affect epiboly driven by radial intercalation. Conversely, Celsr1 dysfunction in teleosts sequentially disrupts both processes (Carreira-Barbosa et al., 2009). Both the radial (this study) and planar organisation (Nishimura et al., 2012) of developing vertebrate organs is dependent upon Celsr1–ROCK signalling, tempting us to speculate that this signalling axis might distinguish or possibly integrate radial and planar cues during vertebrate organ construction.

Taken together, our findings uncover two previously little-known but important stages of mammalian development, namely the primary establishment of the orderly epithelium of the epidermal basal monolayer and the simultaneous spreading of the skin to enclose the embryo body in a protective surface covering. Epidermal epiboly achieved through radial cell intercalation and subsequent planar cell shape changes explains both these processes, but the degree to which they might contribute to ventral closure defects of the abdominal wall, which are poorly understood, will require extensive further investigation.

## MATERIALS AND METHODS

### Mice and staging criteria

The *Celsr1* mouse mutant *Crsh* (BALB/c) and the *fz6-*knockout mouse (C57BL6) have been described previously (Curtin et al., 2003; Guo et al., 2004). All mice were between 8 weeks and 1 year of age. Mice were genotyped by PCR. mT/mG females [Gt(ROSA)26Sortm4(ACTB-tdTomato,-EGFP)Luo/J (Jackson Laboratories strain 007576)] were crossed with a tamoxifen-inducible-Cre male-B6.Cg-Tg(CAG-cre/Esr1*)5Amc/J (Jackson Laboratories strain-004682) to generate mosaic membrane GFP labelling. Wild-type mice for timed explant cultures were outbred CD-1s (in-house). For timed mating, 9am of the first day of plugging was taken as E0.5. Tissue intrinsic morphological criteria were used to determine early epidermal morphogenesis (E13.25). The thickened surface ectoderm was grossly visualised using a dissecting microscope and appeared as a rostro-caudal oriented tissue ridge at the level of the developing forelimb or hindlimb. All animal experiments were performed according to approved guidelines.

### Antibodies and immunohistochemistry

Generation of the Celsr1-specific antibody and immunofluorescence analyses for Celsr1 on frozen sections were as described previously (1:1000; Formstone et al., 2010). Other antibodies were against: cytokeratin 10 (1:1000; Thermo-scientific, RKSE60, MAI-06319), cytokeratin 1 (1:500; Covance, AF109; Hu et al., 2001), cytokeratin 14 (1:1000; Covance, PRB-155P; Roop et al., 1984), cytokeratin 15 (1:500; Millipore, CBL272; Waseem et al., 1999), fibronectin (1:800; Sigma, F3648, 051M4777), E-cadherin (1:4000; DECMA-1, Sigma, U3254; Vestweber and Kemler, 1985), vinculin (1:1000; Sigma, V9131; Geiger et al., 1987), Ki67 (1:100; Abcam, ab16667; Ortiz et al., 2015), phosphorylated histone H3 (1:300; Millipore, 06570; Yamada et al., 2014), frizzled 6 (1:100; R&D systems; Devenport and Fuchs, 2008), myosin II heavy chain (1:500; Sigma, M7659; Wells et al., 1996), myosin light chain (1:50, Cell Signaling, 3671; John et al., 2004), myosin phosphorylated at S19 or S20 (1:500; Rockland, 600-401-416; DiGiovanna et al., 1996), active caspase-3 (1:500; R&D Systems, AF835), and rabbit polyclonal against p63 (1:100; Cell Signaling; Levine, 1997).

Immunofluorescence analyses on frozen sections other than for Celsr1 involved fixing embryos overnight and subsequent incubation in 30% sucrose in PBS until the embryos sank prior to embedding in OCT. Immunostaining was then as for Celsr1. Paraffin sections were stained according to Oudin et al. (2011). Whole-mount immunostaining was essentially as described by Devenport and Fuchs (2008). Sections and whole tissue were imaged on a confocal microscope (Nikon confocal/A1R). Images were analysed using Volocity software (Perkin Elmer).

### Quantitative analyses

Immunohistochemistry was performed on 50-µm thick longitudinal sections. Quantification of oriented cell division was performed by taking sequential images along a stretch of epidermis on a confocal microscope (Nikon-A1R). *z*-stack images were generated using 0.3 µm steps. 3D reconstructions (Volocity-software, Perkin Elmer) of each *z*-stack were generated for each image. All telophase divisions and their underlying basal lamina were individually cropped and the resulting images rotated (3D-opacity mode) until the segregation of each chromatid pair could be measured relative to the basal lamina (marked by staining for fibronectin). One snapshot was taken from different sides of any given division. Angles of cell division relative to the basal lamina were measured using the ruler tool in Adobe Photoshop and the mean angle for each division calculated. In each case, a line was first drawn along the basal lamina and then through the centre of each chromatid, which appeared in side-view (see Fig. 5F). Polar plots were generated in MATLAB. Mitotic cells were scored by morphology and intensity of nuclear DAPI staining and Ki67 staining. Intensity plots were generated with Nikon imaging software*.* For ECM markers, ten separate intensity plots were generated for each region of interest along the dermal/epidermal boundary and the mean values calculated. For cell interface and cell vertex analyses on explants >25 intensity plots were generated for each region of interest and mean values calculated.

### Quantification of epidermal thickness

For frozen sections, epidermis was assessed on both sides of four longitudinal sections and scored for cell depth along their anterior–posterior extent. Individual basal cells sitting on the basal lamina were scored. Local cell thickness was quantified by counting cells overlying each cell sitting on the basal lamina. For whole-mounted epidermis and explants, multiple images of whole-mount immunostained tissue were analysed using Volocity. Different cell groups from different areas of the image were individually cropped and an image snapshot exported into Adobe-Photoshop. The depth of the epidermis was measured using the ruler tool and converted with reference to the scale bar from Volocity.

### Quantification of epidermal cell packing

Multiple images of whole-mount immunostained tissue were analysed using Volocity. Cell packing data were collected by counting nuclei along 100-μm sample lengths in multiple cropped images taken from different areas of the image and at different orientations. For any one image, the number of nuclei in each layer along every 100-μm sample length was scored.

### Quantification of epidermal circumference in the embryo

Contour length of embryonic epidermis from transverse paraffin sections and from images of whole embryos taken on a Leica dissecting microscope was measured in ImageJ.

### Determination of cell proliferation, cell number and cell death

For whole-mount immunostained tissue, the mitotic index was scored using immunostaining for phospho-histone H3 (explants). Cell death index was scored by immunostaining for active caspase-3. The mitotic index and cell death index was calculated for each 1000 basal cells contained within every image analysed. At least three explants from two different litters were analysed or three embryo whole-mounts in each case.

### *Ex vivo* culture

Embryos were dissected from the uterus in HBSS (Sigma) and flank skin dissected away from both sides of the embryo using Lumsden scissors as follows: back skin was first cut along the lateral edge of the spinal cord from the forelimb to the hindlimb. Just inside of the forelimb a 90° turn was executed and the epidermis was cut to just beyond the level of the forelimb. Epidermis was similarly cut from the spinal cord to the just beyond the level of the hindlimb and a rectangular piece of flank skin removed. Tissue intrinsic morphological criteria described above were used to determine the stage of epidermal morphogenesis for dissection purposes.

### Trowell culture

A significant layer of mesoderm was left attached to the epidermis. Explants were immediately placed onto a solid support [PET-track-etched-membrane (Becton-Dickinson) or nucleopore-track-etched-membrane (Whatman)]. Explants cultured on PET-membranes were mounted on a sterile-grid support suspended on a Center-well organ culture dish (Falcon, 35307), overlain with a small amount of epidermal medium [DMEM (Invitrogen) supplemented with fetal bovine serum (10%), 1.8×10^−4^ M adenine (Sigma), 0.5 µg/ml hydrocortisone (Sigma), 5 µg/ml insulin (Sigma), 10^−10^ M cholera enterotoxin (Sigma), 10 ng/ml EGF (Peprotech)] and cultured at 37°C under 5% CO_2_ (Jensen et al., 2010). Nucleopore membranes were floated on top of the epidermal medium and the explant covered by a drop of the same medium. Humidity was maintained using sterile 3 MM paper soaked in sterile PBS. Explants were cultured for 24 h and fixed in 4% formaldehyde in PBS prior to paraffin sectioning.

### Suspension explants

Epidermis and dermis were peeled away from the underlying mesoderm. ‘Peeled’ explants were placed immediately into Lumox dishes (Starstedt) containing the epidermal medium described above. Such explants were free to curl up in culture bringing together the edges, which eventually sealed after 15 h. As a result, the dermis was internalised and covered. Explants were cultured for up to 24 h and fixed in 4% formaldehyde in PBS for either 1 h (whole-mount immunohistochemistry) or 24 h (paraffin sectioning).

### Explant spreading assay

Epidermis was dissected as described for Trowell culture, immersed into a droplet of HBSS, epidermal side up and captured under a glass coverslip secured by two spots of vacuum grease. Slight pressure was exerted to flatten the explant and a bright-field image was taken at low magnification using a Leica dissecting microscope. The glass coverslip was then carefully lifted and the explant transferred onto a membrane support (Trowell culture) or the epidermis was peeled away from the mesoderm and transferred into suspension culture. After the appropriate culture time, Trowell culture explants were lifted into a droplet of HBSS and again captured under a glass coverslip secured by two spots of vacuum grease. Slight pressure was exerted to flatten the explant and a bright-field image was taken at the same magnification as pre-explant. Sharp forceps were used to uncurl Lumox explants (4 h to 10 h) or break into the sealed Lumox explant and open up the tissue (15–24 h). In the latter, the explant was then cut at four equidistant sites from the edge to just inside the centre so that the explant could be opened out flat like a flower with four petals. All explants were lifted into a droplet of HBSS and captured under a glass coverslip secured by two spots of vacuum grease. Slight pressure was exerted to flatten the explant and a bright-field image was taken at the same magnification as pre-explant. For all explants, surface area was measured by measuring the cumulative width and lengths of rectangular sections (ruler tool, Adobe Photoshop) of each explant image both prior and subsequent to culture. For the timecourse experiment, at each time point the change in surface area of several explants was measured (*n*=4) and each explant individually subjected to whole-mount immunostaining to label cell membranes (E-cadherin), mitotic index (phospho-histone H3) and cell death (activated caspase-3).

### Inhibition experiments

Stock solutions of Jasplakinolide (Sigma), cytochalasinB (Sigma), Y27632 (Stemgent), Blebbistatin (Sigma) and CK666 (Tocris) were made in DMSO. RGD and RGE peptides (Takara-Bio-Europe, France) were resuspended in PBS and added to the culture media to the final concentrations described.

### Live imaging of Lumox explants

Mosaic mGFP was induced by injecting pregnant females with 1.7 mg Tamoxifen with progesterone 2 days prior to explanting. Explants were dissected as described above and allowed to develop in Lumox culture for 3–7 h. At the appropriate time, individual explants were gently pushed under a glass coverslip secured with vacuum grease and immobilised by a gentle pressure on the coverslip which slightly flattened the explant. A *z*-stack image was taken at 25× magnification (water dipping objective) using a Leica SP5 laser scanning confocal microscope. The explant was gently moved away from under the coverslip and allowed to develop further without constraint. This process was repeated at a second time point. To register the same group of cells over time a defined area of the explant was marked with DiI on the explant surface. Successful *z*-stack images of these cells were cropped in the *xy* and *z* planes to register the superficial cells, which remained at the tissue surface, and superimposed using Imaris8.0 3D imaging software (Bitplane AG, Switzerland). Individual cell shapes in the merged image were generated manually using each optical section of the *z*-stack and the surfaces function in Surpass view (Imaris). The centre of the nuclear mass for each fluorescent cell was concurrently measured by identifying the *z*-position of the top and bottom of the nuclear space and calculating the central point. The *z*-position was measured from the top of the superimposed *z*-stack image. Statistical analysis was the paired *t*-test.
